# Comparing two distinct costing models for health economics of heart failure within a Swedish population-based cohort

**DOI:** 10.1093/ehjopen/oeag172

**Published:** 2026-10-15

**Authors:** Björn Agvall, Anders Halling, Håkan Nilsson, Awais Ashfaq, Pia Johansson

**Affiliations:** Department of Research and Development, Region Halland, 301 80 Halmstad, Sweden; Center for Primary Health Care Research, Department of Clinical Sciences, Malmö, Lund University, 202 13 Malmö, Sweden; Center for Primary Health Care Research, Department of Clinical Sciences, Malmö, Lund University, 202 13 Malmö, Sweden; Department of Research and Development, Region Halland, 301 80 Halmstad, Sweden; Department of Research and Development, Region Halland, 301 80 Halmstad, Sweden; School of ITE, Halmstad University, 301 18 Halmstad, Sweden; Health Economics and Policy (HEPER), School of Public Health and Community Medicine, Institute of Medicine, Sahlgrenska Academy, University of Gothenburg, 405 30 Göteborg, Sweden

**Keywords:** Heart failure, Healthcare costs, Healthcare resource utilization, Inpatient care, Primary care

## Abstract

**Aims:**

To compare two healthcare costing models, Cost Per Patient and Patient Encounter Costing, in patients with heart failure, quantify differences in estimated healthcare costs, and identify factors associated with discrepancies between the models.

**Methods and results:**

A retrospective register study was conducted in Region Halland, southwest Sweden, including 3230 patients aged 40–90 years with heart failure diagnosed at least twice between 2013 and 2018 and alive during 2019. Healthcare utilization costs during 2019 were estimated using the Cost Per Patient and Patient Encounter Costing models. Linear regression analyses were used to identify factors associated with costs and differences between the models. The Cost Per Patient model estimated a mean annual cost of €10 341 compared with €9538 for the Patient Encounter Costing model. Hospital care accounted for 73% of total costs in both models. Although overall cost estimates were similar, differences in outpatient and primary care costs reached 22%. Higher costs in both models were associated with diabetes, elevated N-terminal pro B-type natriuretic peptide levels, and impaired kidney function. Differences between the models were primarily explained by primary care visits, inpatient care days, and N-terminal pro B-type natriuretic peptide levels. Sensitivity analyses using log-transformed costs yielded similar results.

**Conclusion:**

Both Cost Per Patient and Patient Encounter Costing methodologies were useful for estimating healthcare costs in heart failure. The models provide different perspectives on healthcare costs because they use different cost-allocation principles. These differences should be considered when selecting a costing methodology for healthcare evaluations.

## Introduction

Heart failure (HF), affecting roughly 2% of the Western population, is a serious health condition characterized by diminished functional capacity and quality of life, alongside elevated healthcare utilization and mortality rates.^1–4^ It imposes a substantial economic burden on society, primarily due to the costs associated with hospitalizations and readmissions. One-third of the patients experienced a cardiovascular-related readmission within 100 days and approximately 20% within 30 days.^5–7^ Implementing preventive measures that reduce the necessity for hospital admissions among individuals with HF can substantially lower healthcare expenses, without compromising their quality of life.^8^ The highest cost has been shown to accrue during the first year after onset of HF, at a cost of €12000, while earlier studies reported a cost of up to €7700 annually in year 2005.^8–11^ Another study with data from 2016 reported a cost of €9800.^4^

Healthcare decision-making relies on accounts of healthcare costs. These costs are compared across different years, clinics, disease areas, hospital and primary care settings, and patient groups, as well as treatment modalities. While costs reflect resource utilization patterns, they are influenced not only by the quantities of resources consumed but also by the prices assigned to those resources. The methods used to price healthcare resources thus impact decision-making, potentially leading to biased decisions. The estimation of healthcare costs is typically performed through two main methodologies.^12–14^ One is the activity-based costing method, also known as the cost per patient (CPP) model, and the other is the time-driven activity-based costing, which includes its variant, the patient encounter costing (PEC) model. The distinction between these methods lies in how the resources consumed are priced.

The CPP methodology remains central in health economics in Sweden, providing a fundamental framework for analysing and managing the financial aspects of patient care.^15^ The Swedish National Board of Health and Welfare has collected and reported Swedish CPP data since 2002.^16^ This data is also utilized to estimate weights for the Swedish Diagnosis-Related Groups. The Swedish Diagnosis-Related Groups system exemplifies activity-based costing, which is also the most common approach internationally.^17^

Alternative pricing methods are currently in use or being piloted in Swedish healthcare regions. One such method is time-driven activity-based costing that was introduced as part of the Value-Based Health Care framework, which requires only two parameters: The annual cost of resources involved in care delivery and the amount of time those resources are available each year.^18^ Recent international studies have shown time-driven activity-based costing to be an accurate costing method that supports better-informed quality improvement initiatives.^14,19–22^ It has also proven feasible for integrated multidisciplinary healthcare processes for patients with multiple comorbidities and complex healthcare needs, such as those with heart, nephrology, and diabetes conditions.^23^

A similar costing approach is the PEC model, used within Region Halland.^24^ This approach was developed in collaboration with Harvard Business School.^25,26^ The PEC is applied across all healthcare sectors in Region Halland, including inpatient, emergency, outpatient specialist care, primary care, and ambulance services. These PEC costs have previously been used to study treatment issues, such as those related to HF and chronic kidney disease.^4,27^

Health economic accounts have been proven feasible with the PEC methodology, but direct comparisons between the PEC and CPP costing models are lacking. Because healthcare cost estimates are commonly used for budgeting, benchmarking, resource allocation, and health economic evaluations, differences between costing methodologies may influence both healthcare decision-making and research findings. The extent and implications of such differences remain largely unknown in Swedish healthcare.

The objective of this study was to compare the Cost Per Patient and Patient Encounter Costing models in heart failure, quantify differences in estimated costs across healthcare settings, and identify factors associated with discrepancies between the models.

## Method

This study presents a retrospective, non-interventional observational analysis of healthcare utilization costs among HF patients residing in Region Halland, located in southwest Sweden, with approximately 340 000 inhabitants. The healthcare infrastructure in the region includes three acute care hospitals, 40 inpatient wards, two emergency departments, 30 outpatient specialty clinics, and 46 primary care clinics. Of the primary care clinics, 23 are privately managed, while the others are publicly operated.

### Data source

Region Halland benefits from extensive access to pseudo-anonymized data via the Regional Healthcare Information Platform (RHIP). This dataset encompasses clinical, operational, and financial details for every individual treated since 2011 across all healthcare facilities within the region. Serving as the cornerstone for this study. RHIP's comprehensive dataset spans primary care, emergency department care, hospital admissions, hospital outpatient care, and inpatient care. The database contains examinations, including laboratory, physiological, and radiological tests. It offers a comprehensive view of the complete patient population in Region Halland, merging clinical, operational, and resource use data at the individual patient encounter level, alongside resource and capacity metrics, e.g. the number of full-time equivalent nurses/physicians and hospital bed occupancy rates. RHIP includes information on deceased patients, such as the date of death, facilitating comprehensive all-cause mortality analysis within the cohort. Data regarding dispensed and picked-up pharmacotherapies were obtained from the Swedish National Drug Registry.

### Study population

The study cohort includes patients diagnosed with ICD-10 codes I50 (Heart failure) or I42 (Cardiomyopathy) at least twice, with a minimum interval of 10 days between diagnoses, spanning from 1 January 2013, to 31 December 2018. Participants in the study were aged between 40 and 90 years, with their age assessed as of 1 January 2019. To be eligible for inclusion in the study, patients needed to be alive and have residency in Region Halland at any time during the year 2019.

### Study process

The main study period was the year 2019, with the collection of comorbidity data spanning from 2013 to 2018, as detailed in Supplementary material online, *Table S1*. Additional demographic information, including age and gender, was also gathered. During 2019, laboratory tests to measure estimated glomerular filtration rate (eGFR) and N-terminal pro B-type natriuretic peptide (NT-proBNP) levels were conducted, providing insights into renal and cardiac function, respectively. The date of death for deceased patients and the number of days they were alive in 2019 were recorded. Patients who died during 2019 remained included in the cohort and contributed healthcare utilization until the date of death, ensuring that end-of-life healthcare use occurring during the study year was included in the analyses.

Throughout 2019, individual-level data were collected on a range of healthcare interactions, including inpatient and outpatient hospital care, as well as primary care. The data collected on inpatient care included both the aggregate days of hospitalizations and the frequency of hospital admissions. Outpatient hospital care data were categorized by the type of service, covering visits to the emergency department, consultations with physicians, nursing services, and interactions with paramedical staff. For primary healthcare, the data detailed consultations with physicians and nurses, alongside visits to paramedical staff. Additionally, the number of home visits conducted by physicians and nurses in the primary care setting was documented. Pharmacotherapies were delineated based on selected prescriptions obtained from the pharmacies, encompassing medications specifically targeted for HF, as outlined in Supplementary material online, *Table S2*. The cost estimates presented in *Table 2* were calculated from healthcare utilization during 2019, including hospital days and healthcare encounters, and are reported as mean annual costs for the study population.

### Health economic calculation methodologies

The CPP calculated costs were based on the expenses incurred by clinics, with an allocation of resources to individuals who had any form of medical encounter. The study included individual-level healthcare resource use costed by the two costing methods CPP and PEC, reported in average euros (€) during the year 2019 (SEK 1 vs. €10.6). All costs were estimated using 2019 price levels. Because all healthcare utilization and cost calculations originated from the same calendar year, no adjustment for inflation was required. Purchasing power parity adjustment was not performed as the study did not involve comparisons between different countries or healthcare systems. It focused exclusively on the costs of healthcare providers, including expenses related to patient care, such as inpatient hospitalization, medical procedures, diagnostic tests, laboratory tests, imaging, pharmacotherapies, and both physical and virtual hospital outpatient and primary care encounters. Patient medical healthcare records from the RHIP were used to assign costs according to CPP and PEC to individual patients based on the specific treatments and services they received.

The CPP methodology was used to estimate costs of care per patient based on the type of contact. Costs that were related to a specific contact were allocated to that specific contact. Costs that could be allocated to a service consumed by a contact were assigned to the relevant service and then, in a second stage, allocated to the contact based on the extent of service utilization. The remaining costs were distributed by dividing the total residual costs by the number of contacts performed, and the result was added to the cost of each contact. The process is displayed in detail in Supplementary material online, *Table S3*.

The PEC methodology used the same approach to allocate costs directly related to contacts and services. In the PEC method, one key type of capacity was defined for each type of contact. The remaining costs per contact were then calculated by dividing the total residual costs by the number of contacts that could be performed at the maximum capacity of the key resource and adding the result to the cost of each contact. When analysing inpatient care costs, the key capacity used was the number of available hospital bed-days. For outpatient, emergency, and primary care, the key capacity was the available time of key healthcare personnel. The required capacity information was obtained from operational data within RHIP, including staffing resources, available clinical time, and hospital bed capacity, rather than from investigator-derived assumptions. The process is shown in detail in Supplementary material online, *Table S3*.

These two models exhibited methodological differences, as CPP calculated the cost of a patient encounter by dividing the total expenses of a healthcare unit by the number of patient encounters, while PEC calculated costs per patient encounter without including the cost of vacant care places. For example, in inpatient care, costs are assigned based on the number of available hospital bed-days rather than the total number of hospital stays. In outpatient visits, costs are distributed according to the available consultation time of healthcare providers instead of simply dividing by the number of visits. Similarly, in emergency care, costs are allocated based on the department’s capacity rather than on individual patient interactions. The details of the CPP and PEC calculations are compared and described in the Supplementary material online, *Table S3*.

Non-healthcare provider (indirect) costs such as those associated with sick leave, reduced work capacity, early retirement, costs for other sectors such as local municipal authorities, and patient travel expenses were omitted in both models.

### Statistics

Descriptive statistical methods were employed to detail the attributes of the study cohort, such as age, gender, comorbidities, lab test results, and medication usage. Continuous variables were summarized using the mean and standard deviation (SD), while categorical variables were expressed in terms of frequency and percentage. The Student’s *t*-test was utilized for comparing continuous variables, while for comparisons across multiple groups, ANOVA analysis was applied.

The distribution of healthcare costs across hospital care, primary care, and pharmacotherapy, comparing the two costing approaches, CPP and PEC, showing the percentage breakdown of costs for each care category.

Linear regression analysis was conducted to examine the relationship between disease-related clinical parameters and costs, presented separately for the CPP and PEC models.

Univariate linear regression was used to study the relationship between different costs calculated by CPP vs. PEC. In an additional multivariate linear regression model, additional factors (age, sex, use of health care, eGFR, comorbidities) were added, and a forest plot was generated to illustrate how adding an additional factor explained the difference seen between the unadjusted CPP model and the PEC model. A forest plot was used to visually summarize the regression coefficients and their 95% confidence intervals and to illustrate the relative impact of each additional adjustment factor on the difference between the CPP and PEC models. As healthcare cost data are commonly right-skewed, sensitivity analyses were performed using log-transformed total costs [LN(cost+1)] in multivariable linear regression models. The same covariates were included as in the primary analyses.

All tests were two-sided, and the significance level was set at 0.05. Statistical analyses were conducted using IBM SPSS Statistics version 29 (IBM Corp., 1 New Orchard Road, Armonk, NY, USA), and linear regression was analysed with STATA version 18 (StataCorp, College Station, TX, USA).

## Results

The study included 3230 patients in 2019, with a mean age of 76.5 years. During the study, 357 patients (11%) deceased. The basic characteristics of the study population for 2019 are presented in *Table 1*. The most common comorbidities were hypertension, ischaemic heart disease, and atrial fibrillation. There were 2182 patients (68%) with an elevated NT-proBNP level, while 875 patients (27%) did not have an NT-proBNP analysis during 2019. There were 1891 patients (59%) with decreased renal function, and 2891 patients (90%) had an eGFR measurement during the study period.

**Table 1:** Basic of characteristics of the study population for the year 2019

Variables	
Total cohort, *n*	3230
*Gender*	
Women, *n* (%)	1259 (39)
Men, *n* (%)	1971 (61)
*Age*	
Age, mean (SD)	76.5 (10.1)
40–75, *n* (%)	1275 (40)
>75, *n* (%)	1955 (60)
*Comorbidities*, *n* (%)	
Hypertension	2424 (75)
Ischaemic heart disease	1673 (52)
Cerebrovascular disease	493 (15)
Atrial fibrillation	1851 (57)
Diabetes mellitus	930 (30)
COPD	592 (18)
*Laboratory findings*, *n* (%)	
NT-proBNP, mean (SD)	5832 (10734)
HF not likely	273 (8)
Greyzone	647 (20)
HF likely	1435 (44)
eGFR, mean (SD)	52.5 (18.4)
eGFR >60	1000 (31)
eGFR 30–60	1526 (47)
eGFR <30	365 (11)

Note: *n*, numbers; COPD, chronic obstructive pulmonary disease; NT-proBNP, N-terminal pro b-type natriuretic peptide (ng/L); eGFR, estimated glomerular filtration rate (mL/min/1,73 m^2^).

On average during the year, the cohort had 4.8 hospital days and 7.8 outpatient visits to physicians, nurses, and paramedical staff. The corresponding figure for primary care was 14.7 encounters on average. *Table 2* presents a comparison of the costs calculated using CPP and PEC for each cost item. The percentage difference between the two methods is most pronounced for outpatient care encounters, including both hospital outpatient care and primary care.

**Table 2:** Healthcare services resource use, including hospital inpatient care, outpatient, primary care encounters, and difference in costs according to costing models CPP and PEC, and an overview of the pharmacotherapy costs

				Absolute difference	95% CI of the difference		Difference (%)
	Encounters	CPP	PEC		Lower	Upper	
***Hospital care***							
Inpatient care, mean days (SD)	4.8 (10.3)	5012 (1130)	4712 (1119)	300	271	331	6%
Emergency department	1.0 (1.6)	502 (937)	494 (901)	8	8	9	2%
*Outpatient care*							
Physician	2.2 (3.1)	885 (1699)	791 (1633)	94	84	106	12%
Nurse	2.8 (11.5)	973 (6130)	819 (5729)	154	135	172	19%
Paramedical staff	1.8 (4.8)	148 (551)	117 (451)	31	24	37	26%
***Primary care***							
Physician	3.7 (3.8)	506 (536)	419 (445)	87	84	90	21%
Nurse	4.4 (8.8)	385 (791)	316 (648)	69	64	74	22%
Paramedical staff	6.6 (9.6)	327 (681)	268 (556)	59	55	64	22%
**Total costs healthcare utilization**		8740 (14513)	7937 (13616)	803	762	845	10%
*Pharmacotherapy*							
Cardiovascular		205 (462)					
Diabetes		251 (1067)					
Respiratory		1050 (3725)					
Additional pharmacotherapy		95 (245)					
**Total pharmacotherapy cost**		1601 (4024)					
**Total costs**		10341(11541)	9538 (14656)	803	762	845	8%

Note: CPP, cost per patient; PEC, patient encounter costing; CI, confidence interval; SD, standard deviation; Total pharm cost, total pharmacotherapy cost.

Hospital care accounted for 73% of the total costs, regardless of whether the CPP or PEC model was used. Within this proportion, inpatient care accounted for 48% under the CPP model and 49% under the PEC model. Outpatient care at the hospital represented 24% of costs in the CPP model and 23% in the PEC model. Primary care costs amounted to 12% in the CPP model and 11% in the PEC model. Pharmacotherapy accounted for 15% of the costs in the CPP model, rising to 17% in the PEC model. *Figure 1* illustrates the distribution of costs for hospital care, including inpatient care, outpatient care, and emergency department visits, as well as for primary care and pharmacotherapy across the CPP and PEC models.

**Figure 1:**
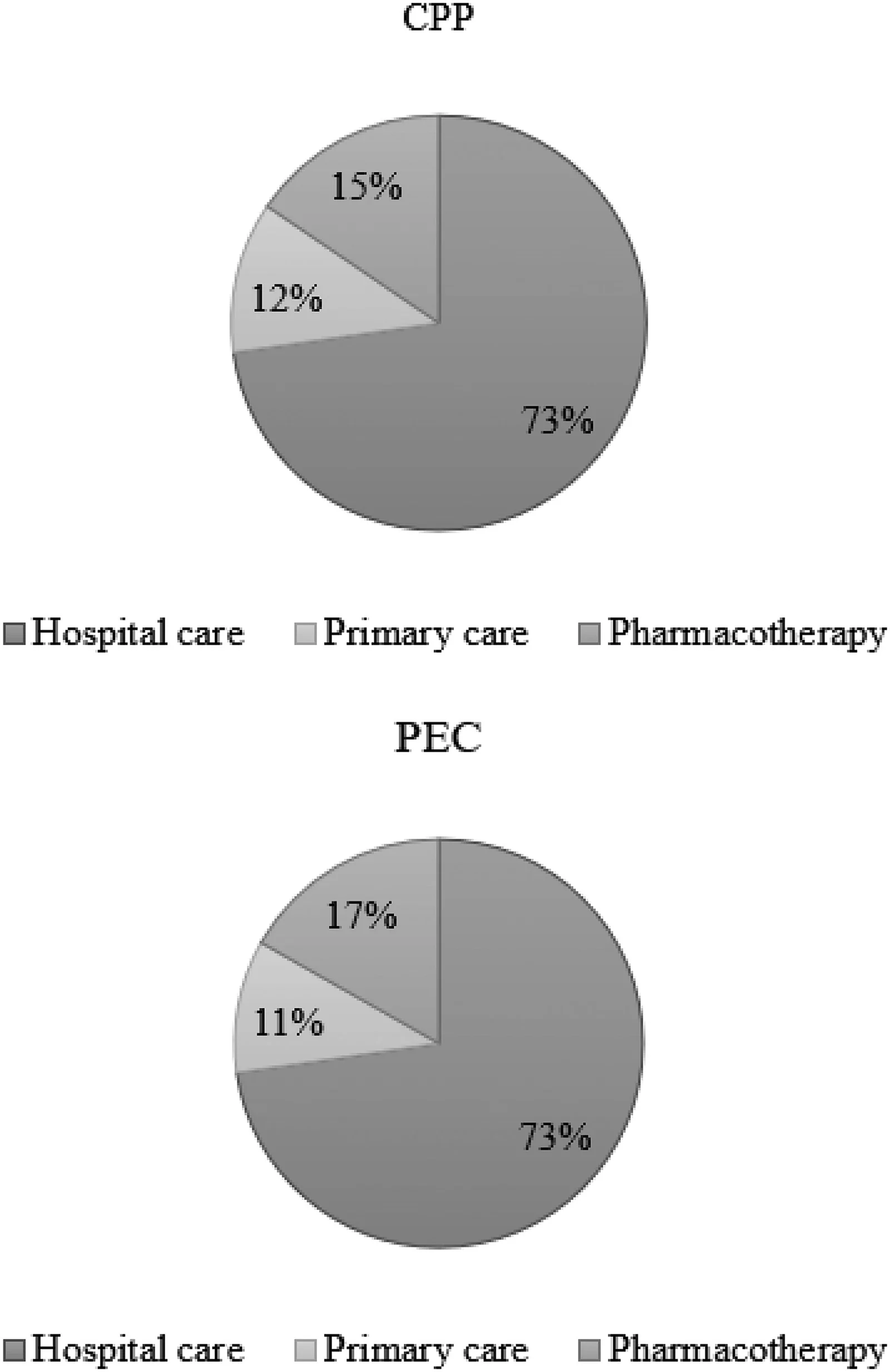
Displays the distribution of costs as percentages across hospital care, primary care, and pharmacotherapy according to CPP and PEC. Note: CPP = cost per patient, PEC = patient encounter costing.

The cost of HF was significantly influenced by increased age (*P* < 0.001), the presence of diabetes (*P* = 0.01), elevated NT-proBNP levels (*P* < 0.001), and reduced eGFR (*P* < 0.001) in the linear regression analysis. These findings were consistent across both cost models in comparable linear regression analysis, which is presented in detail in *Table 3*.

**Table 3:** Linear regression analysis of disease-related clinical parameters and their influence on costs, displayed separately for the cost per patient and patient encounter costing models

	Unstandardized coefficients *B*	95.0% confidence interval for B			
		Lower bound	Upper bound	*P*-value	VIF
*Cost per patient model*					
Men	−167.63	−1459.50	1124.24	0.80	1.06
Age	−141.76	−216.38	−67.15	<0.001	1.46
Hypertension	607.19	−924.90	2139.27	0.44	1.15
IHD	517.68	−748.49	1783.84	0.42	1.06
Atrial fibrillation	−7.29	−1323.03	1308.45	0.99	1.10
Diabetes mellitus	1934.49	537.52	3331.45	0.01	1.07
COPD	1350.16	−155.97	2856.30	0.08	1.01
NT-proBNP	0.38	0.32	0.44	<0.001	1.15
eGFR	−113.11	−154.78	−71.43	<0.001	1.55
*Patient encounter costing model*					
Men	−147.27	−1363.60	1069.05	0.81	1.06
Age	−123.55	−193.80	−53.30	<0.001	1.46
Hypertension	621.48	−821.02	2063.98	0.40	1.15
IHD	372.35	−819.78	1564.47	0.54	1.06
Atrial fibrillation	−85.56	−1324.36	1153.24	0.89	1.10
Diabetes mellitus	1746.88	431.60	3062.16	0.01	1.07
COPD	1353.03	−65.04	2771.09	0.06	1.01
NT-proBNP	0.36	0.31	0.42	<0.001	1.15
eGFR	−102.56	−141.79	−63.32	<0.001	1.55

Note: IHD, ischaemic heart disease; COPD, chronic obstructive pulmonary disease; NT-proBNP, N-terminal pro B-type Natriuretic Peptide; eGFR, estimated glomerular filtration rate.

Age, eGFR, and NT-proBNP were continuous variables, and Sex, IHD, COPD, and AF were categorical variables.

Sensitivity analyses using log-transformed total costs [LN(cost+1)] yielded similar findings. Diabetes mellitus, NT-proBNP, and eGFR remained significantly associated with costs in both the CPP and PEC models (all *P* < 0.001). Furthermore, ischaemic heart disease, atrial fibrillation, and COPD were significantly associated with costs in both log-transformed models (CPP: *P* = 0.021, *P* = 0.016, and *P* = 0.024, respectively; PEC: *P* = 0.027, *P* = 0.016, and *P* = 0.013, respectively).

Comparison between CPP and PEC indicates minimal differences in costs due to inpatient care and emergency department visits, as illustrated by the box plots in *Figure 2*. There are more sizable variations in outpatient care encounters at the hospital and in primary care visits. The costs reported by the CPP model are consistently higher.

**Figure 2:**
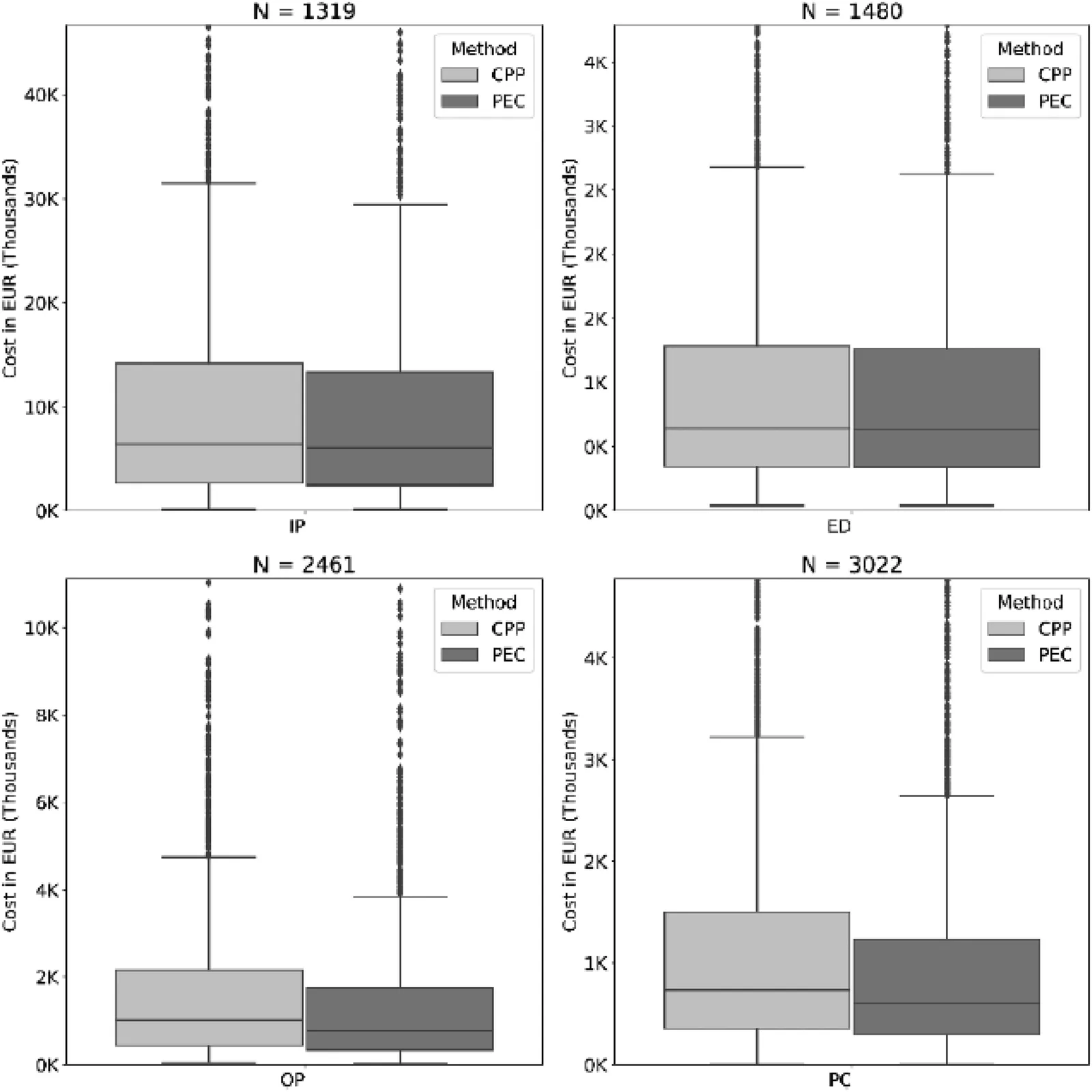
Box-plot models present the costs according to costing models, excluding individuals with no costs during the study period. Note: *N* = numbers, CPP = cost per patient, PEC = patient encounter costing, IP = inpatient care, ED = emergency department, OP = outpatient hospital care, PC = primary care.

*Figure 3* displays a forest plot of the linear regression analyses comparing the CPP and PEC models regarding primary care visits, emergency department visits, inpatient care days, clinical parameters (eGFR and NT-proBNP), specific comorbidities such as diabetes and ischaemic heart disease, and demographic factors including sex and age. The costs in the PEC model were 6% lower than the unadjusted costs in the CPP model. The differences in costs between the CPP and PEC models were affected by the number of primary care visits, inpatient care days, and elevated levels of NT-proBNP.

**Figure 3:**
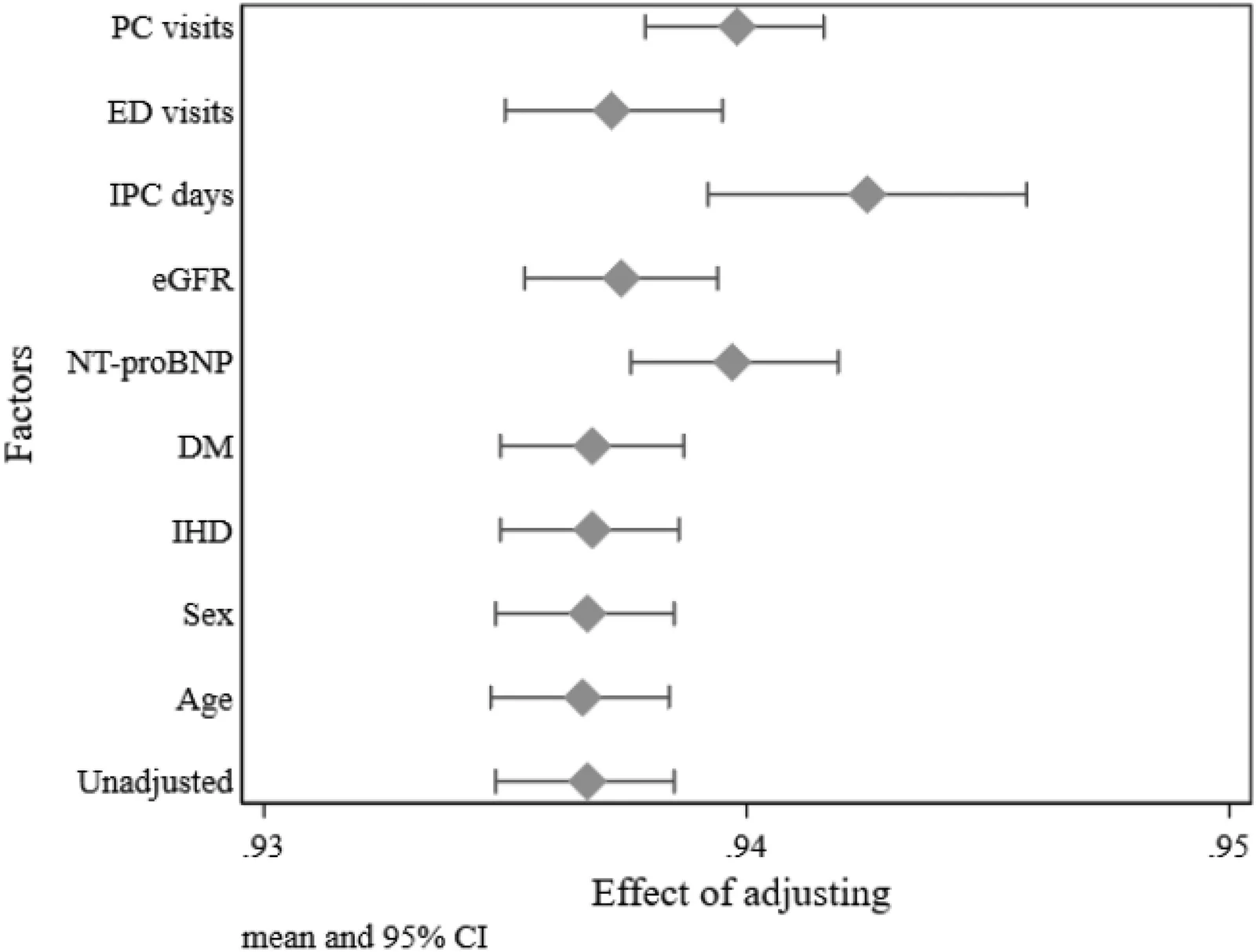
A forest plot of a linear regression comparing the unadjusted CPP with the PEC model. The plot shows the changes in the linear regression coefficient as each additional factor is added to the unadjusted model one at a time. Note: PC visits = primary care visits, ED visits = emergency department visits, IPC days = inpatient care days, eGFR = estimated glomerular filtration rate (categorized), NT-proBNP = N-terminal pro b-type natriuretic peptide (categorized), DM = diabetes mellitus, IHD = ischaemic heart disease.

## Discussion

This study compared two costing models and observed a 6% difference in patient-level costs. The variations were small for inpatient care resource use but reached up to 22% for hospital outpatient and primary care resources, indicating that the choice of costing methodology may influence the results of health economic analyses. The study suggests that the CPP method reflects service costs based on the organization's expenses at a given time, without considering resource efficiency. In contrast, the PEC method estimates costs at the encounter level by allocating residual costs according to available capacity, thereby reducing the influence of short-term variations in capacity utilization. The differences between the two cost models were driven by increased NT-proBNP, primary care visits and inpatient hospital care.

Both the CPP and PEC models have been recognized as useful for calculating healthcare costs, but they have not been directly compared in the context of a costly chronic disease such as heart failure.^23,25,26^ As expected, hospital care, including both inpatient and outpatient services, was the primary cost driver, contributing to three-quarters of the total costs, consistent with previous research.^4,8,27^ This comparison demonstrated that while both models are useful, some discrepancies exist between them. Differences in estimated costs were mainly observed in primary care and hospital outpatient care, whereas inpatient care showed greater consistency between the models. The findings suggest that CPP and PEC may be suited for different purposes. CPP reflects costs incurred by the healthcare organization under prevailing operating conditions and may therefore be appropriate for budgeting, financial planning, and analyses of organizational expenditure. PEC allocates residual costs according to available capacity and may therefore be useful for benchmarking, analyses of resource utilization, and comparisons across healthcare providers or care pathways. Neither method can be considered universally preferable. The choice of costing methodology should therefore be determined by the purpose of the analysis and the perspective adopted. As differences between the models were most pronounced in outpatient and primary care settings, despite relatively modest differences in overall costs, the choice of costing methodology may influence the results of health economic analyses. For hospital administrators and payers, CPP may be preferable when estimating actual organizational expenditure, whereas PEC may be advantageous when comparing efficiency and resource use across healthcare providers or care pathways. The PEC methodology relies on detailed information regarding healthcare encounters, resource availability, and capacity utilization. Although the specific implementation is unique to Region Halland, the underlying principles may be applicable in other healthcare systems with access to similar operational and financial data. Further refinement of costing methodologies in these healthcare settings may therefore improve the consistency of cost estimates.

The costs in both the CPP and PEC models followed similar patterns and were similarly affected by HF-related clinical factors apart from increased levels of NT-proBNP, as demonstrated by the linear regression analysis. When considering costs in both models and clinical parameters, factors such as comorbidities, reduced kidney function, and elevated NT-proBNP were associated with higher costs. In the present study, diabetes, elevated NT-proBNP, and decreased eGFR were all associated with increased costs, with no differences between the CPP and PEC models. Elevated NT-proBNP was associated with greater differences between the costing models, possibly because it reflects more advanced HF and higher healthcare utilization. Since healthcare costs are often right-skewed, sensitivity analyses using log-transformed total costs were performed. Diabetes mellitus and NT-proBNP remained positively associated with costs, whereas lower eGFR remained negatively associated with costs in both costing models. These findings support the robustness of the primary analyses. It can be assumed that these factors were linked to a greater need for hospital care, as shown in previous research. However, in this study, they did not influence the cost differences between the CPP and PEC models.^5,6^

In this cost analysis of patients with HF, the total annual average cost ranges from €9538 to €10 341 according to the costing model, which is comparable to previous analyses using the PEC model and slightly lower than comparable recent studies using CPP and PEC.^4,10^ This study included patients aged 40–90 years with HF diagnoses documented in at least two instances, aiming to more precisely attribute costs to HF. This focus is due to the high comorbidity often seen in this patient group. Hospital care remains the dominant expenditure, while the relatively high number of outpatient and primary care visits generate smaller expenses. Given that the prevalence of heart failure (HF) in Sweden is around 2%, there are approximately 200 000 individuals living with the condition.^27–31^ With an average annual cost of about €10 000 per patient, the total direct healthcare costs for HF patients amount to roughly €2 billion.

### Strength and limitations

A strength of the study is its detailed scrutiny of an entire region and its extensive healthcare system. While some healthcare may occur outside the region, this is regarded as minimal. Consequently, the study offers a unique and comprehensive set of variables for each individual included. Furthermore, our study serves as an example of an explicit comparison between pricing models and the resulting cost estimates. The study compares two costing methodologies but does not include an external reference standard, which prevents conclusions regarding the absolute accuracy of either costing approach. Furthermore, the PEC implementation requires detailed operational and financial data on healthcare encounters, staffing resources, time available for clinical care, and hospital bed capacity. Such data may not be readily available in all healthcare systems, which may limit the direct transferability of the approach. NYHA classification was not included in the analyses because these data were not available with sufficient completeness and consistency within the RHIP database. NT-proBNP was therefore used as an objective and routinely collected marker of HF severity.

In this study, the inclusion criteria are stringent, requiring patients to have had at least two HF diagnoses according to ICD-10. Additionally, there is an age restriction to address similar concerns. Patients under 40 are rare, while those over 90 often have significant comorbidities and frequently do not receive treatment due to being in terminal stages. The study has limitations in that it cannot establish causal conclusions and can only highlight associations.

### Conclusion

This study compared two costing models among HF patients, CPP and PEC, revealing a 6% difference in patient-level costs, with variations up to 22% for outpatient and primary care. Both models are viable but serve different purposes. CPP reflects the costs incurred by the healthcare organization under prevailing operating conditions, whereas PEC estimates encounter-level costs using a capacity-based allocation approach. NT-proBNP, inpatient care, and primary care visits influenced the cost differences, but other HF parameters did not. The potential changes in principles for cost allocation and resource pricing need to be considered when a new costing model is introduced.

## Supplementary Material

oeag172_Supplementary_Data

## Data Availability

The datasets generated and analysed during this study are not publicly available, in accordance with the Swedish Health and Medical Services Act and the Secrecy Act. However, these datasets may be requested from Region Halland through the corresponding author. Access will be granted following a review by the Regional Consultative Committee for data collection in Region Halland.
