# Supplementary material for: Comparing two distinct costing models for health economics of heart failure within a Swedish population-based cohort
Source: Eur Heart J Open. 2026 Oct 15;6(5):oeag172. doi: 10.1093/ehjopen/oeag172 (PMC13717912; doi:10.1093/ehjopen/oeag172)
Supplement: Supplementary file 1 [file oeag172_supplementary_data.docx]

**Suppementary Information**

**Supplementary Table 1**

Supplementary Table 1. ICD-10 codes for heart failure and the comorbidities included in present study

| **Disease** | **ICD-10 codes** |
| --- | --- |
| Heart failure (head diagnosis) | I42, I50 |
| Hypertension | I10 - I15 |
| Ischemic heart disease | I20 - I25 |
| Cerebrovascular insult | I60 – I69 |
| Atrial fibrillation | I48 |
| Diabetes mellitus | E10 – E14 |
| Chronic obstructive pulmonary disease | J44 |

**Supplementary Table 2**

Supplementary-Table 2: List of evidence-based medications recommended for treatment of heart failure.

**Medication (ATC-code)**

Angiotensin-converting-enzyme inhibitors (C09)

Captopril

Enalapril

Lisinopril

Ramipril

Trandolapril

Betablockers (C07)

Bisoprolol

Carvedilol

Metoprolol

Nebivolol

Angiotensin receptor blockers (C09)

Candesartan

Valsartan

Losartan

Mineralocorticoid receptor antagonists (C03DA)

Eplerenone

Spironolactone

**Supplementary Table 3**

| **Cost Per Patient Model** | **Patient Encounter Costing Model** |
| --- | --- |
| **Identify Total Healthcare Costs:** Sum all healthcare-related costs within the specified period. | **Identify Total Healthcare Costs:** Sum all healthcare-related costs within the specified period. |
| **Allocate Direct Costs:** Assign all costs that can clearly be linked to a specific patient contact directly to that contact. | **Allocate Direct Costs:** Assign all costs that can clearly be linked to a specific patient contact directly to that contact. |
| **Allocate Service-Related Costs:** Assign costs that can be linked to a specific healthcare service to that service. Then, distribute the cost to patient contacts based on their use of that service. | **Allocate Service-Related Costs:** Assign costs that can be linked to a specific healthcare service to that service. Then, distribute the cost to patient contacts based on their use of that service. |
| **Determine Remaining (Unassigned) Costs:** Subtract the total of direct costs and service-related costs from the total healthcare costs. The remaining amount represents other costs not linked to specific contacts or services. | **Determine Remaining (Unassigned) Costs:** Subtract the total of direct costs and service-related costs from the total healthcare costs. The remaining amount represents other costs not linked to specific contacts or services. |
| **Determine the Number of Contacts:** Count the number of contacts performed for the patients who received care during the specified period. | **Determine the Maximum Number of Contacts at Full Capacity:** Estimate the maximum number of patient contacts that could occur during the period under full operational capacity. |
| **Distribute Remaining Costs:** Divide the remaining unassigned costs by the number of contacts to calculate the additional cost per contact. | **Distribute Remaining Costs:** Divide the remaining unassigned costs by the maximum number of contacts to calculate the additional cost per contact. |
| **Calculate Final Cost per Patient Contact:** Add the direct cost, service-related cost and additional cost per contact. | **Calculate Final Cost per Patient Contact:** Add the direct cost, service-related cost and additional cost per contact. |
| **Calculate cost per patient:** Add the cost of all contacts per patient to calculate total patient cost. | **Calculate cost per patient:** Add the cost of all contacts per patient to calculate total patient cost. |

Supplementary Table 3. A step-by-step allocation of costs for each healthcare cost calculation model followed by an example scenario.

**Heart Failure Example Scenario**

Consider a hospital or healthcare center that provides care for patients with heart failure. Over a 1-month period, the following costs are incurred:

1. Total healthcare organization costs for the heart failure patient group: €120,000
2. Direct costs (hospital admissions, consultations, etc.): €50,000
3. Service-related costs (e.g., diagnostic tests, medications, imaging, etc.): €40,000
4. Remaining unallocated (overhead) costs: €30,000
5. Number of unique heart failure patients treated: 80
6. Maximum number of patient contacts possible at full capacity (considering follow-up visits, admissions, and consultations): 150
7. In this simplified example each patient has 1 contact

*Cost Per Patient Model*

Step-by-step calculation:

1. Total healthcare costs: €120,000
2. Direct costs for patient contacts: €50,000
3. Service-related costs for patient contacts: €40,000
4. Remaining unallocated costs: €30,000
5. Calculate remaining costs per contact
   Remaining unallocated costs: €30,000 ÷ 80 (contacts) = €375 per contact
6. Final cost per patient contact:
   Average* direct costs (€50,000 ÷ 80 contacts = €625 per contact)
   Average* Service-related costs (€40,000 ÷ 80 contacts = €500 per patient)
   - Total cost per contact = Direct cost + Service-related cost + Remaining overhead
     €625 + €500 + €375 = €1,500 per contact
   - Total cost per patient with 1 contact = €1,500

*Patient Encounter Costing Model*

Step-by-step calculation:

1. Total healthcare costs: €120,000
2. Direct costs for patient contacts: €50,000
3. Service-related costs for patient contacts: €40,000
4. Remaining unallocated costs: €30,000
5. Calculate remaining costs per patient encounter:
   Remaining unallocated costs: €30,000 ÷ 150 (max possible contacts) = €200 per contact
6. Final cost per patient contact:
   Average* direct costs (€50,000 ÷ 80 contacts = €625 per contact)
   Average* service-related costs (€40,000 ÷ 80 contacts = €500 per contact)
   - Total cost per patient contact = Direct cost + Service-related cost + Remaining overhead
     €625 + €500 + €200 = €1,325 per contact
   - Total cost per patient with 1 contact = €1,325
7. Total patient encounter cost = €1,325 x 80 contacts for 80 patients with 1 contact each = €106,000

*Key Comparison and Insights*

- Cost Per Patient Model results in a higher cost per patient (€1,500) when capacity utilization is less than 100 percent, here the overhead costs are distributed across fewer contacts (80).
- Patient Encounter Costing Model distributes the overhead costs across a greater number of contacts (here 150 possible encounters), resulting in a lower per-contact cost of €1,325.
- The patient encounter cost for the same type of contact will not vary just due to variation in capacity utilization over time.

Normally each contacts exact direct cost and cost of service consumed are assigned to each contact. These costs are not calculated using averages as in this simplified example used only to illustrate the difference between the two cost calculation models.
